# A Review of the Catalytic Mechanism of Human Manganese Superoxide Dismutase

**DOI:** 10.3390/antiox7020025

**Published:** 2018-01-30

**Authors:** Jahaun Azadmanesh, Gloria E. O. Borgstahl

**Affiliations:** 1 Department of Biochemistry and Molecular Biology, 985870 Nebraska Medical Center, Omaha, NE 68198-5870, USA; jahaun.azadmanesh@unmc.edu; 2 Eppley Institute for Cancer and Allied Diseases, 986805 Nebraska Medical Center, Omaha, NE 68198-6805, USA

**Keywords:** superoxide dismutase, human, mechanism, anti-oxidant, reactive oxygen species, mitochondria, manganese, catalysis

## Abstract

Superoxide dismutases (SODs) are necessary antioxidant enzymes that protect cells from reactive oxygen species (ROS). Decreased levels of SODs or mutations that affect their catalytic activity have serious phenotypic consequences. SODs perform their bio-protective role by converting superoxide into oxygen and hydrogen peroxide by cyclic oxidation and reduction reactions with the active site metal. Mutations of SODs can cause cancer of the lung, colon, and lymphatic system, as well as neurodegenerative diseases such as Parkinson’s disease and amyotrophic lateral sclerosis. While SODs have proven to be of significant biological importance since their discovery in 1968, the mechanistic nature of their catalytic function remains elusive. Extensive investigations with a multitude of approaches have tried to unveil the catalytic workings of SODs, but experimental limitations have impeded direct observations of the mechanism. Here, we focus on human MnSOD, the most significant enzyme in protecting against ROS in the human body. Human MnSOD resides in the mitochondrial matrix, the location of up to 90% of cellular ROS generation. We review the current knowledge of the MnSOD enzymatic mechanism and ongoing studies into solving the remaining mysteries.

## 1. Introduction

Superoxide (O_2_•^−^) is a potent oxidizing agent. Excessive amounts lead to a cascade of reactions causing damage to important biological macromolecules such as DNA, lipids, and proteins. Excess superoxide plays a role in the pathogenesis of many disease states including cancers, cardiovascular disorders, and neurodegenerative diseases [1,2,3]. To protect cells from harmful amounts of superoxide, SODs convert two superoxide anions to oxygen and hydrogen peroxide using a cyclic reduction and oxidation reaction of the active site metal. This redox shuffling of the active site metal to perform catalysis, called dismutation, is dependent on two protons per cycle.

$$\begin{array}{l} {\mathrm{Mn}}^{3+}+O_{2}^{•-}↔{\mathrm{Mn}}^{2+}+O_{2}\\ {\mathrm{Mn}}^{2+}+O_{2}^{•-}+2H^{+}↔{\mathrm{Mn}}^{3+}+H_{2}O_{2} \end{array}$$

The coordinated metals at the active site classify SODs. FeSOD and NiSOD are prokaryotic whereas FeSOD is also present in chloroplasts. CuZnSODs are located in the extracellular matrix and cytosol of eukaryotes. MnSOD dwells within bacteria and all eukaryotes. Of note, FeSODs and MnSODs have near identical active site configurations [4]. In eukaryotes, MnSOD is in the mitochondrial matrix, an organelle compartment with a high rate of endogenous superoxide generation. Electrons leak from the electron-transport chain and perform a one-electron reduction of diatomic oxygen to form superoxide [5]. The ability of MnSOD to decrease superoxide levels in the mitochondria is associated with longevity of eukaryotic organisms [6]. MnSOD knockout mice die within the first day of life due to dilated cardiomyopathy and neurodegeneration whereas overexpression of MnSOD in fruit flies increases lifespan [7,8]. Sequence comparison and conservation of the active site residues among all published MnSOD structures shows all MnSODs have identical active sites [9]. In humans, polymorphisms of MnSOD have been associated with type 2 diabetes, hypertension, and the onset of prostate cancer [10,11]. Due to its necessary antioxidant role in biological systems, characterization of the catalytic mechanism of MnSOD has been the goal of many research groups. However, the atomic mechanism has been elusive because of (1) the fast reaction rate of MnSOD; (2) the high reactivity and short half-life of superoxide; and (3) the difficulties in detecting protons and protonation states of amino acids in an enzyme [12,13]. This review describes the current knowledge of the MnSOD mechanism and ongoing studies, with a focus on the human variant for its medical relevance. Several articles review other SOD mechanisms [4,14,15].

## 2. Medical Relevance and Therapeutics

The presence of excessive superoxide and its derivatives, such as hydroxyl radical (OH•), peroxynitrite (ONOO^−^), and nitrogen dioxide (NO_2_), leads to oxidative stress and is prevalent during inflammation, mitochondrial degeneration, and tumorigenesis [16]. Chronic inflammation can lead to disease states such as atherosclerosis and type two diabetes whereas mitochondrial degeneration leads to neurological disorders such as cerebral palsy and Parkinson’s disease. Cells undergoing oxidative stress are susceptible to transformation and metastasis in a wide variety of cell types [17]. This is partly due to superoxide acting as a signaling molecule to induce cell division and proliferation [18]. Articles of this special issue discuss the roles of oxidative stress, superoxide, and SODs in cancer [19,20,21], and further reviews describe how oxidative stress influences disease states [22,23,24,25]. Therapeutic approaches in pre-clinical and clinical studies have arisen from the ability of MnSOD to decrease levels of superoxide and mitigate oxidative stress. There are three types of therapeutics: (1) MnSOD as an administered drug; (2) MnSOD expression as a transgene; or (3) as a small molecule mimetic [16,26,27].

Using MnSOD as a drug has been appealing due to its bio-protective role and used over other SODs because Mn is not susceptible to Fenton chemistry like Fe and Cu. MnSOD administration has been tested for irradiation protection, immunosuppression for transplantations, reduction of inflammation, and fibrosis in vivo in rats and humans [28,29,30]. Of note, MnSOD use in pre-clinical studies using rats and mice are applicable to humans due to high sequence identity, with 93% and 94% conservation, respectively. While using MnSOD for treatments has shown promising results, practical issues arise from the short half-life of the enzyme (6 min) due to rapid renal clearance [31]. Protein engineering to increase longevity of MnSOD may overcome this issue [32,33]. An alternative method for increasing life-span is delivery via liposomes, which augments the half-life to 4 h [31]. While MnSOD is a powerful protectant against diseases, its use as a drug requires chronic administration.

Development of gene therapy-based delivery approaches for MnSOD hopes to circumvent the issues of its short half-life. These investigations aim to increase MnSOD levels by production within cells. MnSOD overexpression by vector delivery showed significant toxicity for tumor cells and radio-protective properties for healthy cells in vitro and in vivo in mice [34,35,36]. Tumors cells have deficient systems for tolerating hydrogen peroxide production from dismutation whereas healthy cells are tolerant. Human clinical trials are ongoing for utilizing MnSOD expression from a plasmid containing liposome to confer radioprotection to healthy cells during concurrent radiotherapy and chemotherapy treatment for non-small-cell lung cancer [26]. Further details of using MnSOD for therapeutic approaches can be found in a detailed review by Borrelli and colleagues [27].

An alternative to utilizing the MnSOD enzyme is to use a small molecule mimic. The most promising mimics are Mn porphyrins, resembling the Fe containing heme group. Other classes of mimics are discussed in a review by Miriyala and colleagues [16]. Mn porphyrins are attractive because they (1) lack antigenicity; (2) are extremely stable; (3) can penetrate subcellular membranes; (4) scavenge other ROS such as peroxynitrite; and (5) are modifiable to optimize efficacy for their desired approach, all while approaching the catalytic rate and efficiency of MnSOD [37]. Mn porphyrin mimetics MnTE–2–PyP^5+^ and MnTnBuOE–2–PyP^5+^ have shown promising results in irradiation protection of healthy cells during radiotherapy and for treating diabetes, as noted within articles of this special issue [38,39] and in phase I/II clinical trials [40,41].

As expected, the design principles of MnSOD mimetics used to achieve a high catalytic rate are derived from mechanistic studies of MnSOD, which include a configured electrostatic guidance, coordinated water ligands, and fine-tuned redox potential [42,43]. However, the means by which superoxide interacts with the mimetics and substrate protonations are not clear. Further understanding the details of MnSOD catalysis has the potential to optimize efficacy of mimetics and protein engineered constructs for treatment approaches.

## 3. Function of Active Site Residues

**Figure 1:**
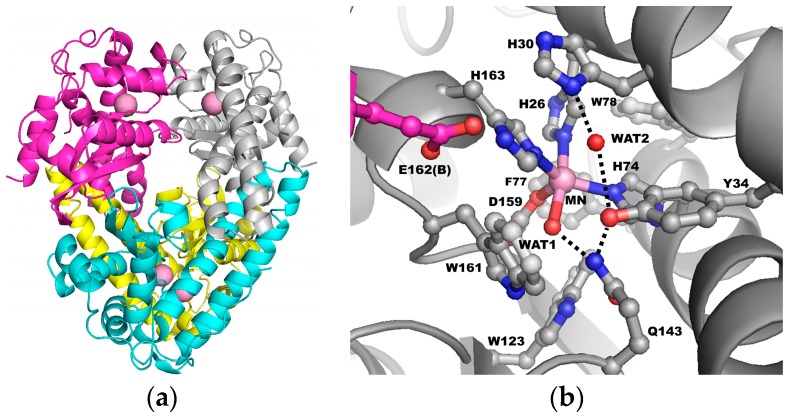
Human MnSOD. (**a**) Each subunit contains a manganese ion at the catalytic center, indicated by pink spheres. (**b**) The active site. Red spheres denote oxygen atoms, blue denotes nitrogen atoms, grey denotes carbon atoms from one subunit of the tetramer, and magenta denotes carbon atoms from the adjacent subunit. The dashed lines represent the hydrogen bond network hypothesized to be the proton relay to the manganese ion used for catalysis. Glu162 hydrogen bonds with His163 across the dimer interface. Adapted from Azadmanesh et al., 2017, PDB entry 5VF9 [44]. WAT1: single oxygen-containing molecule; WAT2: single oxygen-containing molecule. Single letter amino acid code is used.

Human MnSOD functions as a homotetramer, with each subunit containing an active site surrounding a manganese ion (Figure 1a). The metal is coordinated by His26, His74, His163, Asp159, and a single oxygen-containing molecule (denoted WAT1), thought to be either H_2_O or OH^−^ (Figure 1b) [44]. These amino acids and ligands, termed the “inner sphere” residues, form a direct interaction with the manganese. The next layer of contacting amino acids, called the “outer sphere” residues, are essential for efficient dismutation. These are His30, Tyr34, Phe77, Trp78, Trp123, Gln143, Trp161, and from across the dimer interface, Glu162 [45,46,47,48,49,50]. Each of these residues plays a role in catalysis.

The outer sphere residues work together to promote the extremely fast catalysis of MnSOD. Substrate is thought to diffuse into the active site through residues His30 and Tyr34 where it most likely binds to the manganese ion in the position opposite Asp159 [44]. The ~5 Å gap between His30 and Tyr34 is the only solvent-accessible area that allows entry into the active site (occupied by a single oxygen-containing molecule, denoted WAT2 in Figure 1b). Residues of the outer sphere, Phe77, Trp78, Trp123, and Trp161, form a hydrophobic cage opposite the solvent-accessible portion of the active site to promote substrate interaction with the manganese ion [45]. Glu162 from the adjacent subunit hydrogen bonds with His163 to stabilize oligomerization [50]. Tyr34, His30, Gln143, and two single-oxygen containing molecules (denoted WAT1 and WAT2) form a hydrogen-bond network that is thought to serve as a proton relay to the manganese ion for proton-assisted electron transfer (dashed lines, Figure 1b) [46,47,48,49,51]. Investigations into the catalytic mechanism have been unable to determine the path of proton transfers owing to limitations in hydrogen detection.

## 4. Substrate Diffusion to the Active Site

MnSOD has one of the fastest and most efficient reaction rates of all enzymes, with a *k*_cat_ of 40,000 s^−1^ and a *k*_cat_/*K_M_* close to 10^9^ M^−1^ s^−1^ [52]. Given that superoxide is a negatively charged substrate, MnSOD probably achieves rapid catalysis with the aid of electrostatic guidance. In 1983, Getzoff and colleagues were the first to calculate electrostatic field vectors for a SOD. For bovine Cu/ZnSOD, they found that superoxide guidance to the active site is a long-range process [53], which means that neutralization of a charged amino acid far from the active site, such as acetylation of a lysine, would perturb the net field vectors and hamper guidance of the substrate to the active site [44].

**Figure 2:**
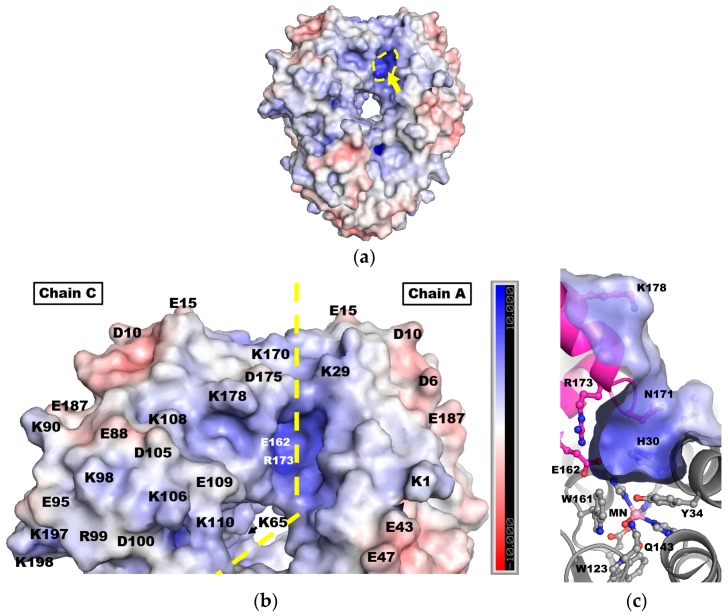
Electrostatic surfaces of human MnSOD. (**a**) The electrostatic surface of the tetramer with only one active site viewed in this orientation (yellow arrow and dashed lines). (**b**) A zoomed-in view of the dimer interface indicated by a yellow, dashed line. The labels for Glu162 and Arg173 are white to indicate their location on the concave surface within the active site pit. (**c**) A cross section-view of the active site across the dimer interface, rotated 90° along the horizontal axis in relation to (**a**,**b**). Glu162 is behind Arg173 in this view. Electrostatic surface is colored in kiloteslas. Adapted from Azadmanesh et al., 2017 [44]. Single letter amino acid code is used.

The active site of human MnSOD is within a cavity formed by two adjacent subunits (Figure 2a, arrow). Calculated electrostatic surfaces show that the cavity is positively charged, due to the presence of the manganese cation. Basic residues Lys29, and from the adjacent subunit, Lys170, Lys178, and Arg173 contribute to the positive surfaces near the active site cavity (Figure 2b). Interestingly, acetylated Lys29, Lys65, and Lys98 reduce measured enzyme activity [54,55,56]. At the outer ridge of the protein is a cluster of acidic residues contributing to negative electrostatic surfaces, such as Asp6, Asp10, and Glu15. The enrichment of positive electrostatic surfaces near and at the active site in conjunction with the negative surfaces at the edges of the protein explains the productive diffusion of superoxide to the active site. The negatively charged substrate is repelled from the outer areas of the tetramer and attracted to the positively charged cavity of the active site.

At the active site cavity there is an interesting pair of oppositely charged amino acids. Residues Glu162 and Arg173, are 7 and 12 Å from the active site manganese, respectively (Figure 2c). Efficient enzymatic activity requires this negative and positive pair. MnSODs, FeSODs, and CuZnSODs all conserve these residues. Mutation of Glu162 to alanine or aspartate in human MnSOD decreases enzymatic activity 5–25%, and increases product inhibition (discussed later) two-fold. While the Glu162Asp mutation maintains the charge, the side chain is one carbon shorter and breaks a hydrogen bond at the dimer interface, resulting in a packing defect. This decreased stability at the dimer interface may account for the activity loss [50]. Chemical modification of Arg173 with phenylglyoxal abolishes activity, suggesting a crucial role for a positive charge at this site [57,58]. Getzoff and colleagues studied the influence of the negative and positive amino acid configuration in the active site cavity of bovine CuZnSOD (consisting of Glu131 and Lys134) [53]. These amino acids direct the net positive electrostatic vectors into the active site, with mutations of either amino acid changing the net vector direction by at least 20 degrees. Glu131 also reduces non-productive association of a passing superoxide molecule with Lys134. These studies show that Glu162 and Arg173 that line the active site cavity play a significant role in productive entry of the substrate to the active site.

## 5. Superoxide Binding and Active Site Geometry

How superoxide interacts with the catalytic site has been difficult to investigate owing to the short half-life and high reactivity of superoxide in solution. Crystallographic and spectroscopic studies have instead used azide (N_3_^−^) as a substrate analog to study superoxide binding [59,60,61]. The azide anion is a potent competitive inhibitor of SODs and binds directly to the active site metal [62,63]. Of note, azide and superoxide are different enough in size that their binding to the active site could differ. Regardless, studies with azide suggest several mechanisms.

**Figure 3:**
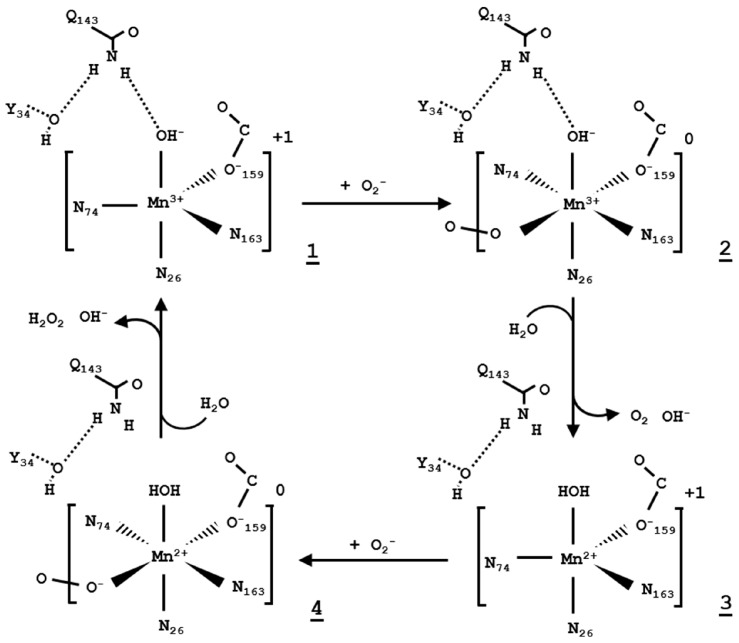
Human MnSOD 5-6-5 mechanism adapted from Lah et al., 1995 [59]. Dotted lines indicated hydrogen bonds.

Lah and colleagues outlined a binding mechanism based on an azide-bound *Thermus thermophilus* MnSOD crystal structure (Protein Data Bank (PDB) entry code 1MNG) where the azide molecule binds to a sixth coordinate position opposite to the metal-bound aspartate. They propose that the resting state of the MnSOD active site is five-coordinate distorted trigonal bipyramidal which shifts to six-coordinate octahedral upon superoxide binding (Figure 3) [59]. Widening of the angle of two adjacent histidine-ligands (His74 and His163) accommodates binding opposite of the aspartate-ligand. *Caenorhabditis elegans* and human MnSOD crystal structures with azide bound also support this mode of binding (PDB entry 5AG2 and 5T30) [44,64]. A 100 K *Escherichia coli* MnSOD crystal structure at alkaline pH (8.5), in which the enzyme is inactive, shows a hydroxide anion at the sixth-coordinate position (PDB entry 1D5N) [65]. This further suggests that this position is the location of superoxide binding at the active site. However, so far no one has structurally captured the location of the highly reactive superoxide anion in the active site. The 5-6-5 mechanism describes the active site manganese dynamically changing its coordination during dismutation.

Whittaker and Whittaker proposed the “associative displacement” mechanism from their thermochromism (temperature-dependent optical spectra) studies of *E. coli* MnSOD with azide [66,67]. The associative displacement mechanism is an alternative mode of superoxide binding, defined by a five-coordinate manganese ion in both the resting and substrate-bound forms. Their findings suggest that the active site of *E. coli* MnSOD remains five-coordinate trigonal bipyramidal at physiological temperature (295 K). Upon substrate binding, an unidentified manganese ligand displaces, with protonated aspartate or the solvent molecule being the most likely candidates. At low-temperatures (275–280 K), the coordination becomes six. These spectroscopic observations are in conflict with the six-coordinate azide-MnSOD structure from *T. thermophilus* (PDB 1MNG), where the crystallization and data collection was at room temperature [59]. Since *T. thermophilus* thrives in relatively high temperatures (320–350 K), room temperature may be a “cold” temperature for the thermophilic species. Whittaker and Whittaker do note that the six-coordinate complex is only marginally unstable at 295 K and suggest it could act as a kinetic intermediate [66,67]. Thus, the mechanism of superoxide dismutases studied with optical spectra suggests a differing mode of superoxide-substrate binding.

Density function theory (DFT) calculations performed by Jackson and co-workers with *E. coli* MnSOD indicate that the active site with azide adducts exists in a dynamic equilibrium between five and six coordinate states at 296 K. At 273 K, the coordination shifts to predominately six [68]. However, the active site states of either coordination were not supportive of displacement of metal bound ligands, indicating that these calculations are not favorable for the associative displacement mechanism. Instead, the authors propose that the superoxide substrate may convert to products without coordination to the metal. This could occur through hydrogen-bond interactions with Tyr34. In nuclear magnetic resonance (NMR) studies of *E. coli* FeSOD by the Miller group, azide did not bind to the reduced form of the active site metal, but instead was near Tyr34. These studies indicate that direct binding of superoxide to the active site metal for catalysis does not occur for at least some portions of the enzymatic mechanism [69].

## 6. Proton Transfers and the Hydrogen Bond Network

For MnSOD to perform its enzymatic function efficiently it must shuttle protons to the active site for proton-assisted electron transfer in a systematic manner. Extensive investigations through both experimental and theoretical approaches studied the proton-based mechanism and yielded several conflicting catalytic models [14,51,59,65,67,68,70,71,72,73,74,75,76,77]. The lack of consensus from the multitude of interdisciplinary approaches is a consequence of the experimental difficulty of directly detecting protons.

To date, insight into the proton-based mechanism has come from indirect observations. X-ray diffraction does not detect the hydrogen atom well, but analysis of X-ray crystal structures reveal a hydrogen bond network at the active site, consisting of His30–WAT2–Tyr34–Gln143–WAT1 (hereafter referred to as the superoxide-independent network, Figure 1b). This configuration is conserved in all Mn and FeSODs [78] and is thought to be involved in a proton relay for proton-assisted electron transfer at the active site metal [14,45,46,47,48,50,72,79]. Mutation of His30, Tyr34, or Gln143 disturbs catalysis drastically, indicating the importance of these residues in enzymatic function [14].

Theoretical studies of the proton-shuttling mechanism have been performed through quantum mechanical molecular modeling (QM/MM) and DFT [51,68,70,75,80,81]. Such approaches attempt to address the underlying complexity of how pK_a_s of amino acids and solvent are influenced at the active site to allow systematic proton transfers [82]. The positively charged manganese at the active site lowers the pK_a_s of amino acids and solvent allowing easier deprotonation. Conversely, positively charged ionization would be unfavorable and increase the pK_a_s of molecules with such capacity (protonation). The extent of influence that manganese has on the pK_a_s of molecules at the active site is dynamically changing through shuffling of its redox state (i.e., Mn^3+^ vs. Mn^2+^). pK_a_s are further determined by whether ionization would make a non-covalent interaction more favorable. Together, the net changes in ionization (i.e., proton transfers) at the active site must be energetically downhill and be able to regenerate through cycling of the redox state of the manganese cation.

The metal-bound solvent molecule, WAT1, is probably a redox-linked proton accepter and perhaps the most studied component of the network [70,71,83]. QM/MM and DFT calculations indicate that in the Mn^3+^ state, the solvent ligand is hydroxide (OH^−^), with its ionization stabilized by the electrostatic interaction with Mn^3+^. Upon conversion to the Mn^2+^ state, the solvent ligand is protonated to H_2_O, counterbalancing the changes in the metal charge (Figure 3). The second half reaction regenerates the Mn^3+^–OH^−^ state, with the proton from the solvent ligand likely reducing superoxide to hydroperoxyl ion (HO_2_^−^), which accounts for one of the two protons needed for conversion to H_2_O_2_. The source of the second proton to convert HO_2_^−^ to H_2_O_2_ is poorly understood and is hypothesized to come from His30, WAT2, Tyr34, or bulk solvent [59,70]. Protonation and deprotonation of the solvent ligand likely happens when a superoxide is bound at the active site (not necessarily directly to the manganese) as this makes product disassociation exergonic and enhances product formation [70]. These theoretical studies attempt to clarify the source of the two protons during the enzymatic reaction of MnSOD.

Second sphere residues appear to play critical roles in catalysis. Working backwards from WAT1 in the hydrogen bond network, Gln143 and Tyr34 are evolutionarily conserved and mutation abolishes SOD activity. Yet, their exact functional role is unresolved [14]. Proton shuttling through these amino acids to WAT1 would require their protonation or deprotonation. For Gln143, both possibilities are highly unfavorable in QM/MM calculations [70]. For Tyr34, its protonation (–OH_2_^+^) is chemically unlikely in both QM/MM and DFT but its deprotonation (–O^−^) is plausible [51,70]. However, this deprotonation would interfere with superoxide binding due to electrostatic repulsion. Direct NMR observations by the Miller group have shown that Tyr34 likely remains in the neutral state (–OH) in both oxidized and reduced states of the enzyme but these studies do not take into account substrate binding effects [71]. These investigations suggest the roles for Gln143 and Tyr34 may be limited to orienting and positioning superoxide substrate and the solvent ligand.

**Figure 4:**
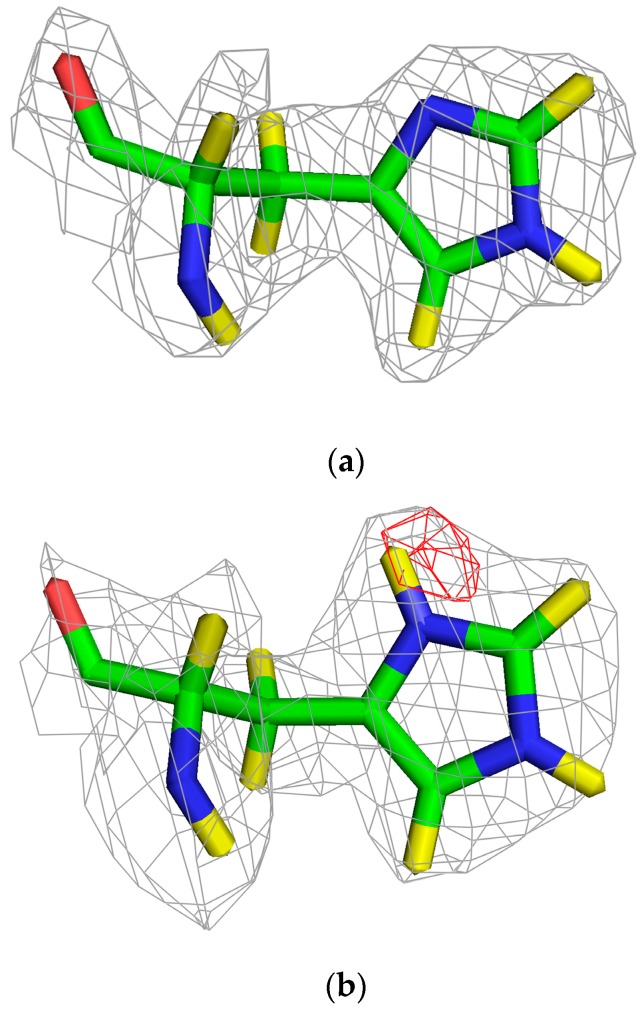
Protonation state of His30 in human MnSOD revealed by neutron diffraction. Carbons are green, nitrogens are blue, and oxygens are red. Deuterium (yellow) replaced hydrogen in the sample to increase diffraction signal. The 2F_o_-F_c_ grey nuclear density is 1.0 σ. The red omit positive F_o_-F_C_ nuclear density is at 2.5 σ. (**a**) Chain A of the neutron crystal structure displays an unprotonated His30. (**b**) Chain B shows a protonated His30. Methods and statistics for crystal growth, deuterium exchange, data collection, and data reduction are found in Azadmanesh et al., 2017 [84]. Data was refined to 2.30 Å using PHENIX.REFINE with *R_work_* and *R_free_* values of 0.28, and 0.31, respectively (unpublished) [85,86].

DFT calculations indicate His30 and WAT2 could relay protons [51]. While protonation to a positive charge is unfavorable at the manganese-containing active site, the net sum of proton transfers is energetically favorable. Borgstahl and collaborators have recently employed the use of neutron diffraction to directly detect hydrogen positions and observed protonation of His30 (Figure 4) [84]. This also suggests that WAT2 can be protonated as it is the nearest hydrogen-bond acceptor/donor to His30 in the crystal structure. The proton shuttling through His30, WAT2 and WAT1 is feasible. It is noteworthy that protonation of His30 would change the electrostatic surface potentials at the active site opening to be more positive (Figure 2c).

**Figure 5:**
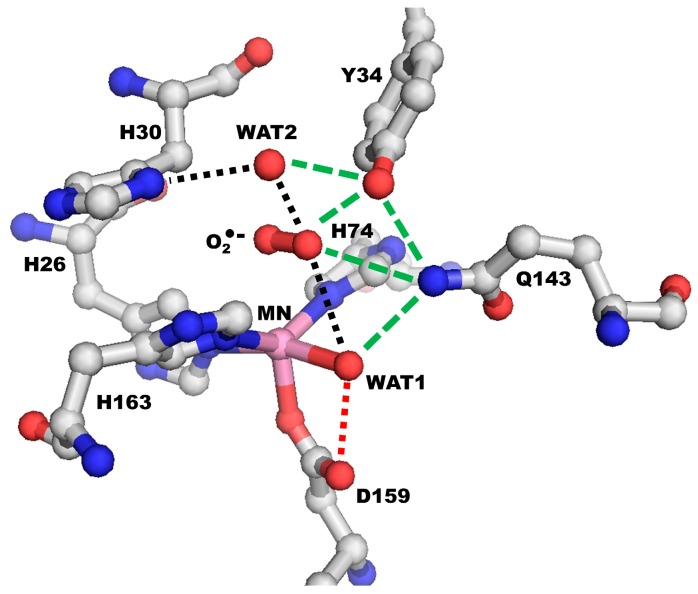
Putative superoxide-dependent proton shuttling mechanism. Black dashed lines indicate the proton transfer relay during catalysis. Red dashed line indicates the proton transfer from WAT1 to Asp159 that results in the inactive complex whereas the backwards transfer restores activity of the enzyme (model based on PDB entry 5T30). Green dashed lines indicate stabilizing hydrogen-bond interactions for molecules of the proton relay. Of note, superoxide is thought to bind Mn^3+^ directly but not Mn^2+^, where it is instead bound to the active site solely through hydrogen bonding to members of the hydrogen-bond network. Single letter amino acid code is used.

A superoxide-dependent proton shuttling network is thought to exist because superoxide binding to the active site allows different hydrogen-bonding and more energetically favorable conditions for proton transfers [51,70] (Figure 5). Such a pathway, His30–WAT2–O_2_•^−^–WAT1 or His30–O_2_•^−^–WAT1, could involve superoxide binding at the active site either directly to the manganese or by non-covalent interactions with Tyr34, Gln143, WAT2, and/or WAT1. QM/MM calculations by Srnec and colleagues suggest that superoxide binding differs between half-reactions, with superoxide directly binding to the manganese in the oxidized state but not in the reduced state [70]. These results are supported by NMR experiments which observe substrate-analog binding to the metal of FeSOD only in the oxidized state [69]. These investigations indicate that superoxide binding to the active site forms an alternative hydrogen-bond network that allows for exergonic proton shuttling.

Studies of combined pulse radiolysis and metal visible absorption spectra experiments have shown that all MnSODs exhibit product-inhibition. An inactive complex can form when manganese is in the reduced state. Here, the superoxide-bound complex, O_2_•^−^–Mn^2+^–(H_2_O), is converted to oxidized peroxide-bound manganese, O_2_^2−^–Mn^3+^–(OH^−^), and peroxide does not disassociate [73,87,88]. In contrast, the active enzyme converts into oxidized hydroperoxyl-bound manganese, HO_2_^−^–Mn^3+^–(OH^−^), and hydroperoxyl radical disassociation is favorable. In both cases, an electron transfer occurs between the manganese and superoxide substrate, with the differences between these two outcomes being the fate of the WAT1 proton. During the active state, superoxide gains a proton from the solvent ligand to form hydroperoxyl radical, whereas the inactive complex forms due to proton donation from the solvent ligand to an alternative proton acceptor. The most probable acceptor is the carboxylate group (–COO^−^) of metal-bound Asp159 as it is within hydrogen-bond distance of the solvent ligand. Protonation of Asp159 would likely lead to disassociation from the metal. Mutagenesis and QM calculations support this inactivation mechanism [89,90]. To regenerate the active enzyme, the proton from the carboxylic acid group (–COOH) of Asp159 is donated back to the WAT1 ligand to reform superoxide-bound reduced manganese, O_2_•^−^–Mn^2+^(H_2_O), in conjunction with an electron transfer between the substrate and the metal. The reaction can then move forward to dismute superoxide.

A cryotrapped hydrogen peroxide-soaked X-ray crystal structure of *E. coli* MnSOD provides three-dimensional visualization of the putative product-inhibited complex (PDB entry 3K9S) [77]. A molecule containing two oxygen atoms is bound side-on to the manganese cation at the active site. This molecule had two differing orientations. However, the resolution of the diffraction data limited the identification of the protonation states of the amino acids and ligated molecules. Oxidized MnSOD in the presence of excess hydrogen peroxide is known to instigate a backwards enzymatic reaction of MnSOD, in which hydrogen peroxide is converted to superoxide [73]. This puts the identity of the double-oxygen containing molecules into question. Hydrogen positions of this structure would be informative in deciphering a product-inhibited mechanism.

Of note, FeSODs, which have identical active site amino acid configurations that are nearly superimposable on those of MnSODs, do not exhibit a reversible inactive, product-inhibited complex [4]. Instead, FeSOD inactivation occurs through the Fenton reaction. Regardless, the catalysis of superoxide into oxygen and hydrogen peroxide products likely follows extremely similar proton-shuttling mechanisms for both Fe and MnSODs.

## 7. Discussion

MnSOD is of great interest to the medical field because of its protection against the deleterious effects of excessive superoxide in disease states [22,23,24,25]. A defense against the pro-oxidant insults of superoxide has brought the potential of treating a plethora of human dysfunctions, including neurodegenerative diseases, diabetes, and cancer within reach [91]. Identifying the multi-step means of catalysis would provide atomic details useful for the design of mimetics and engineered protein constructs for therapeutics.

The MnSOD mechanism includes three categories: (1) the means of highly efficient substrate and product diffusion; (2) the mode of superoxide binding to the active site; and (3) the proton-shuttling mechanism for proton-assisted electron transfer. An intricately fine-tuned electrostatic surface and long-range electrostatic vectors guide superoxide substrate to the active site and products away. Substrate-analog binding provides clues as to how superoxide binds to the active site of MnSOD, yet there is no consensus, with direct or indirect binding to the manganese with or without Mn-ligand displacement being possible modes. The difference in molecular length of superoxide and substrate analogs makes it possible that they do not bind exactly the same way. Two protonations occur during each redox cycle of catalysis that involve rapid catalytic turnover and a high catalytic efficiency [52]. This points to protonations as a particularly orderly process, most likely through a hydrogen-bond relay at the active site. Theoretical calculations point to a proton-relay forming upon superoxide binding to the active site as the most energetically favorable means of proton shuttling [51,70]. From the culmination of experimental and theoretical data, four proton shuttling mechanisms are possible for MnSOD, depending on the stage of catalysis (Figure 5).

(1) $${\mathrm{Mn}}^{3+}+O_{2}^{•-}\to {\mathrm{Mn}}^{2+}+O_{2}\;H^{+}\;\mathrm{from}\;\mathrm{bulk}\;\mathrm{solvent}\to \mathrm{His}30\;\to \mathrm{WAT}2\;\to O_{2}^{•-}\to \mathrm{WAT}1\;\left({\mathrm{OH}}^{-}\right)\;\mathrm{Final}\;\mathrm{proton}\;\mathrm{acceptor}:\;\mathrm{WAT}1\;\left({\mathrm{OH}}^{-}\right)\to \;\mathrm{WAT}1\;\left(H_{2}O\right)$$

(2) $${\mathrm{Mn}}^{2+}+O_{2}^{•-}+2H^{+}\to {\mathrm{Mn}}^{3+}+H_{2}O_{2}\;H^{+}\mathrm{from}\;\mathrm{WAT}1\;\left(H_{2}O\right)\to \;O_{2}^{•-}\;\mathrm{Final}\;\mathrm{proton}\;\mathrm{acceptor}:\;O_{2}^{•-}\to \;{\mathrm{HO}}_{2}^{-}$$

(3) $${\mathrm{Mn}}^{2+}+O_{2}^{•-}\to \;{\mathrm{Mn}}^{3+}+O_{2}^{2-}\;\left[\mathrm{Inactive}\;\mathrm{Complex}\right]\;H^{+}\mathrm{from}\;\mathrm{WAT}1\;\left(H_{2}O\right)\to \mathrm{Asp}159\left({\mathrm{COO}}^{-}\right)\;\mathrm{Final}\;\mathrm{proton}\;\mathrm{acceptor}:\;\mathrm{Asp}159\left({\mathrm{COO}}^{-}\right)\to \mathrm{Asp}159\left(\mathrm{COOH}\right),\;\mathrm{Asp}159\;\mathrm{disassociates}\;\mathrm{from}\;\mathrm{Mn}$$

(4) $${\mathrm{Mn}}^{3+}+O_{2}^{2-}\;\left[\mathrm{Inactive}\;\mathrm{Complex}\right]\to {\mathrm{Mn}}^{2+}+O_{2}^{•-}\;H^{+}\mathrm{from}\;\mathrm{Asp}159\left(\mathrm{COOH}\right)\to \;\mathrm{WAT}1\;\left({\mathrm{OH}}^{-}\right)\;\mathrm{Final}\;\mathrm{proton}\;\mathrm{acceptor}:\mathrm{WAT}1\;\left({\mathrm{OH}}^{-}\right)\to \;\mathrm{WAT}1\;\left(H_{2}O\right),\;\mathrm{Asp}159\;\mathrm{reassociates}\;\mathrm{to}\;\mathrm{Mn}$$

The source of the second proton to convert hydroperoxyl radical to hydrogen peroxide is hypothesized to come from other players of the proton relay, His30 and WAT2, and/or bulk solvent [59,70]. Even 50 years after their discovery by Irwin Fridovich in 1968, the question of how SODs work is still unanswered [92].

## 8. Conclusions

The mechanistic SOD field would benefit from direct observation of the binding mode of superoxide and the proton relay. Observing the manner of superoxide binding has not been possible due to the exceedingly short half-life of superoxide and the fast catalytic rate of SODs. Visualization of protonation states through conventional structural biology techniques is difficult. NMR and X-ray crystallography are not very sensitive to revealing the positions of hydrogen, especially on solvent molecules. Recently, neutron diffraction experiments, which can reveal hydrogen positions, have been employed to investigate human MnSOD [84]. The experiments use a neutron diffractometer tailored to biological samples called the Macromolecular Neutron Diffractometer (MaNDi), which opened for academic use at Oak Ridge National Laboratory in 2014 [93]. It would be intriguing to couple the backwards reaction of the enzyme in the presence of excess hydrogen peroxide with cryo-neutron crystallography. These technological developments are making this experimentally elegant question addressable.
